# Recombinant T-Cell Receptor Ligand (RTL) for Treatment of Multiple Sclerosis: A Double-Blind, Placebo-Controlled, Phase 1, Dose-Escalation Study

**DOI:** 10.1155/2012/954739

**Published:** 2012-04-05

**Authors:** Vijayshree Yadav, Dennis N. Bourdette, James D. Bowen, Sharon G. Lynch, David Mattson, Jana Preiningerova, Christopher T. Bever, Jack Simon, Andrew Goldstein, Gregory G. Burrows, Halina Offner, Al J. Ferro, Arthur A. Vandenbark

**Affiliations:** ^1^Department of Neurology, Oregon Health & Science University, L226, 3181 SW Sam Jackson Park Road, Portland, OR 97239, USA; ^2^Department of Veterans Affairs Medical Center, Portland, OR 97239, USA; ^3^Multiple Sclerosis Center, Swedish Neuroscience Institute, Seattle, WA 98122, USA; ^4^Department of Neurology, University of Kansas Medical Center, Kansas City, KS 66160, USA; ^5^Department of Neurology, Indiana University School of Medicine, Indiana University MS Center, Indianapolis, IN 46202, USA; ^6^Multiple Sclerosis Center, Yale University, New Haven, CT 06510, USA; ^7^Department of Neurology, School of Medicine University of Maryland, Baltimore, MD 21201, USA; ^8^Artielle ImmunoTherapeutics Inc., Tigard, OR 97223, USA; ^9^Department of Anesthesiology & Perioperative Medicine, Oregon Health & Science University, Portland, OR 97239, USA; ^10^Senior Research Career Scientist, Research Service, Medical Center Department of Veterans Affairs Portland VA, Portland, OR 97239, USA; ^11^Department of Molecular Microbiology & Immunology, Oregon Health & Science University, Portland, OR 97239, USA

## Abstract

*Background*. Recombinant T-cell receptor ligand 1000 (RTL1000) is a single-chain protein construct containing the outer two domains of HLA-DR2 linked to myelin-oligodendrocyte-glycoprotein- (MOG-) 35–55 peptide. Analogues of RTL1000 induce T-cell tolerance, reverse clinical and histological disease, and promote repair in experimental autoimmune encephalomyelitis (EAE) in DR2 transgenic, C57BL/6, and SJL/J mice. *Objective*. Determining the maximum tolerated dose, safety, and tolerability of RTL1000 in multiple sclerosis (MS) subjects. *Methods*. This was a multicenter, Phase I dose-escalation study in HLA-DR2^+^ MS subjects. Consecutive cohorts received RTL1000 doses of 2, 6, 20, 60, 200, and 100 mg, respectively. Subjects within each cohort randomly received a single intravenous infusion of RTL1000 or placebo at a 4 : 2 ratio. Safety monitoring included clinical, laboratory, and brain magnetic resonance imaging (MRI) evaluations. *Results*. Thirty-four subjects completed the protocol. All subjects tolerated the 2–60 mg doses of RTL1000. Doses ≥100 mg caused hypotension and diarrhea in 3 of 4 subjects, leading to discontinuation of further enrollment. *Conclusions*. The maximum tolerated dose of RTL1000 in MS subjects is 60 mg, comparable to effective RTL doses in EAE. RTL1000 is a novel approach for MS treatment that may induce immunoregulation without immunosuppression and promote neural repair.

## 1. Introduction

The pathogenesis of MS likely involves increased CD4^+^ T-cell responses directed against myelin antigens, including myelin oligodendrocyte glycoprotein (MOG) [1]. Myelin-reactive T-cells are present even in healthy controls but are activated and occur at higher frequencies in MS subjects [2], due possibly to escape from tolerance mechanisms. An elusive goal for the treatment of MS is the development of therapies that can reestablish tolerance without causing immunosuppression.

Encephalitogenic CD4^+^ T-cells respond to specific myelin peptides complexed with major histocompatibility (MHC) class II molecules on antigen-presenting cells (APC). Ligation of the CD4^+^ T-cell receptor in combination with costimulatory molecules results in activation of autoreactive T-cells that migrate into the central nervous system (CNS) and trigger an inflammatory cascade, resulting in tissue injury and clinical disease. A variety of approaches can block antigen-specific T-cell activation, including intravenous exposure to high doses of free antigen [3], presentation of antigen by modified APCs [4], oral ingestion of antigen [5, 6], or injection of MHC/antigen complexes [7, 8].

Injection of soluble MHC/antigen complexes suppresses clinical and histological signs of experimental autoimmune encephalomyelitis (EAE) [9, 10]. This strategy utilizes an encephalitogenic myelin peptide bound by autologous MHC class II alleles, inducing anergy after T-cell engagement of the soluble MHC/antigen complex in the absence of costimulatory molecules [11]. This approach for treatment of MS became practical by our development of recombinant single chain, two domain MHC class II molecules linked covalently to autoantigenic peptides [12]. These recombinant T-cell receptor ligands (RTLs) proved highly effective for reversing established EAE in several different rodent models [13–16]. To develop an RTL potentially effective for MS, we combined the immunodominant MOG-35-55 peptide with the *β*1-*α*1 domains of HLA-DR2 [17], the highest genetic risk factor that occurs in ~60% of North American and Northern European MS patients [18, 19]. We further demonstrated that this RTL construct, designated as RTL1000, was highly effective at suppressing and treating MOG-35-55 peptide-induced EAE in DR2 transgenic mice [14, 20].

To determine the maximum tolerated dose of RTL1000 in subjects with MS, we performed a multicenter, Phase 1, placebo-controlled, single dose-escalation study. This study demonstrated that a dose of 60 mg of RTL1000 was well tolerated and importantly is comparable to single doses of RTL1000 that are highly therapeutic in DR2 transgenic mice with MOG-35-55-induced EAE.

## 2. Materials and Methods

### 2.1. Subjects

This study was conducted under an FDA approved IND (no. 100128) by Artielle ImmunoTherapeutics, Inc., Tigard, OR, approved by Institutional Review Boards from the six participating institutions (Oregon Health & Science University, Portland, OR; Swedish Neuroscience Institute, Seattle, WA; University of Kansas Medical Center, Kansas City, KS; University School of Medicine, Indiana University MS Center, Indianapolis, IN; Yale University, New Haven, CT; University of Maryland School of Medicine, Baltimore, MD) and registered at http://www.clinicaltrials.gov/ (NCT00411723). All subjects gave informed consent before entering the study. Qualified subjects met the following inclusion/exclusion criteria: definite diagnosis of MS by McDonald criteria [21]; confirmed diagnosis of RRMS or SPMS; age 18–65; Expanded Disability Status Scale (EDSS) of 0.0 to 6.5; no clinical exacerbations within the 8 weeks before administration of study drug; HLA-DR2 positive; not pregnant or breastfeeding and using an acceptable form of birth control; no exposure to any investigational agent or use of recombinant interferon beta, glatiramer acetate, or systemic corticosteroids in the past 4 weeks; no treatment with a monoclonal antibody, natalizumab, or systemic immunosuppressants, including azathioprine, mycophenolate mofetil, methotrexate, cladribine, cyclophosphamide, or mitoxantrone in the past 6 months; no total lymphoid irradiation or bone marrow transplant at any time.

### 2.2. Study Design

This was a multicenter, double-blind, placebo-controlled Phase 1 dose-escalating trial with six consecutive treatment cohorts. The study was designed to enroll six MS subjects per cohort using a ratio of 4 : 2 subjects randomly assigned to receive a single dose of RTL1000 or placebo, respectively. Subjects were admitted to an inpatient research unit, received the study drug by intravenous (IV) infusion over approximately 1 hour (Cohorts 1–4) or 2 hours (Cohorts 5 and 6). Subjects were observed during the infusion and for 24 hrs afterward. To further evaluate safety, subjects were evaluated weekly for 28 days and again on month 3 when they exited the study.

#### 2.2.1. Endpoints

The primary endpoints of the study were safety and determination of the maximum tolerated dose (MTD) of a single IV infusion of RTL1000. The secondary endpoint was to evaluate pharmacokinetics (PK) of RTL1000 in a subset of subjects. Safety laboratory parameters included electrocardiogram (EKG), vital signs, blood chemistries, complete blood count, and antibodies to RTL1000, MOG-35-55 peptide, and HLA-DR2. Clinical safety parameters included medical and neurologic history and examination, EDSS, 25-foot timed walk, 9-hole peg test, gadolinium-enhanced brain MRI, and adverse events. Clinical assessments were performed by site investigators who were masked to the treatment assignment of subjects.

Adverse events and laboratory results were graded according to the common terminology criteria for adverse events, CTCAE v3.0. Subjects were closely monitored for allergic or infusion reactions during the administration of the product. An independent Data Safety Monitoring Board (DSMB), comprised of two neurologists and a statistician, reviewed blinded data at the completion of each cohort and gave permission to initiate enrollment in the next cohort if prespecified safety criteria were met.

#### 2.2.2. Procedure Time Points

 Subjects enrolled in the trial underwent neurological examination and MRI scan at baseline. On Day 0, just prior to infusion of RTL1000 or placebo, blood was drawn and plasma frozen for later evaluation of antibody titers and concentrations of RTL1000. Additional blood was drawn during and immediately after the infusion for PK evaluation of RTL1000 levels in plasma in subjects who agreed to participate in the PK substudy. After completion of the infusion, subjects underwent brain MRI (Day 28), neurological examination (Day 28 and 3 months) and antibody levels (Day 28 and 3 months).

#### 2.2.3. RTL1000 and Placebo

 RTL1000 was supplied as a sterile liquid for IV infusion. Each 10 mL vial contained 10 mg RTL1000 at a concentration of 1 mg/mL in 20 mM Tris buffer at pH8.5. The placebo consisted of Tris buffer solution only, which was visually indistinguishable from the solution with RTL1000. RTL1000 or placebo was infused over 1 hour for doses of ≤60 mg and 2 hours for doses of 100–200 mg. RTL1000 or placebo labeled in a blinded fashion with the subject randomization number was shipped for each subject.

#### 2.2.4. Clinical and Safety Monitoring

 Safety and tolerability were evaluated throughout the study by monitoring subject chemistry and hematology laboratory panels, EKGs, MRIs, neurologic and physical examinations, EDSS, 25-ft timed walk and 9-hole peg test.

#### 2.2.5. MRI Procedures

 Using a standardized procedure, brain MRIs were performed at baseline and Day 28. All study MRIs were screened at the individual study sites for incidental and nonstudy findings. MRIs were transferred electronically to a central reading center at the Portland VA Medical Center under the direction of Dr. Jack Simon. All MRI analyses were performed blinded to treatment allocation. The following assessments were made: total number of gadolinium enhancing lesions on the baseline and D28 scans and new and persistent gadolinium enhancing lesions and new and enlarging T2 hyperintensities on the D28 scan. The frequency of subjects with active scans (defined as those with ≥1 gadolinium enhancing lesions) in each cohort was also determined on the baseline and D28 MRI.

### 2.3. Assessment of Immunosuppression

Six subjects agreed to participate in an immunology substudy. For these subjects, peripheral blood mononuclear cells (PBMCs) were collected prior to infusion of drug or placebo and at 14 and 28 days after infusion and were stored in liquid nitrogen. Samples from each time point were cultured and analyzed as a group for reactivity to anti-CD3 mAb. Briefly, 250,000 PBMC were cultured in triplicate wells in RPMI 1640 with 1% pooled human serum in the presence of 1 *μ*g anti-CD3 mAb or buffer control. Culture supernatants were collected after 48 h and sent to AssayGate, Inc. (Ijamsville, MD) for analysis of IL-1*α*, IL-6, IL-8, IL-10, IL-12(p40), IL-15, IL-17, IP-10, MCP-1, MIP-1*α*, MIP-*β*, and TNF-*α*.

### 2.4. Statistical Analyses

#### 2.4.1. Study Conduct, Baseline Characteristics, and Safety

Descriptive statistics were used to summarize study conduct, baseline measures, and safety (including RRMS disease parameters). Adverse events were tabulated by dose cohort, system organ class, preferred term, according to frequency, severity, and investigator-determined relationship to study drug. Basic descriptive statistics for antibody O.D.s including mean ± SD were carried out for each sample collection time point, and *P* values were derived by applying Fisher's exact test comparing the ratio of positive subjects receiving RTL1000 versus placebo at month 1 or month 3 versus baseline.

## 3. Results

### 3.1. Trial Profile

**Figure 1:**
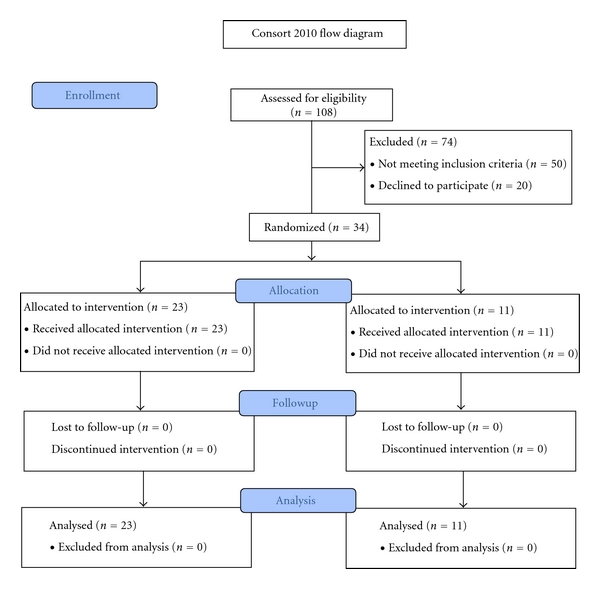
Flow chart of subject enrollment and treatment. One hundred eight MS subjects were screened, with 67 testing positive for expression of HLA-DR2. Of these, 29 failed additional screening (did not meet EDSS requirement, were taking exclusionary drugs, or had a surgical procedure) or declined entry and 38 were enrolled in the trial.

 Between January, 2007, and November, 2008, after signing the informed consent, 108 MS subjects were initially assessed for eligibility and screened for HLA-DR2 (Figure 1). Of these, 50 did not meet inclusion criteria and 20 declined to participate. Of the remaining 38 subjects, 34 were randomized as described (Table 1). Four subjects who met entry criteria were not randomized as the study was stopped before they were randomized. All 34 treated subjects completed the protocol.

Upon the recommendation of the DSMB, Cohort 2 was repeated because one subject receiving 6 mg study drug developed chest pain. No adverse events were encountered with a second cohort (2A) receiving the same dose. Cohort 5, which received 200 mg, was stopped because two of the three subjects receiving study drug experienced significant infusion-related adverse events. With permission of the DSMB, Cohort 6 was initiated to receive an intermediate dose (100 mg), but treatment of this cohort was stopped after the first subject, who received study drug, experienced adverse events similar to those observed in Cohort 5.

### 3.2. Safety and Maximum Tolerated Dose

#### 3.2.1. Adverse Events

 No serious adverse events occurred during the study. RTL1000 infusions were well tolerated at doses of 60 mg or less. The overall incidence of adverse events was similar in subjects receiving RTL1000 versus placebo (87.0% RTL1000, 81.8% placebo). In subjects receiving RTL1000 at doses of 60 mg or less, adverse events did not differ between subjects receiving study drug and placebo aside from the occurrence of chest pain in one subject receiving 6 mg in Cohort 2. This subject experienced chest pain during the infusion that resolved and did not delay discharge; the event was assessed as treatment related by the site investigator; no cardiac or pulmonary etiology was found, despite extensive in-hospital workup. Chest pain did not occur in other subjects receiving RTL1000. Dose-limiting adverse events occurred in subjects receiving doses above 60 mg. One subject receiving 100 mg of RTL1000 had nausea, vomiting, diarrhea, headache, chills, and decreased blood pressure. Two of the three subjects who received 200 mg of RTL1000 experienced similar reactions, and these two subjects also experienced tachycardia, fever, and an increased neutrophil count. All events resolved within 24 hr and discharge from the inpatient research unit was not delayed. Based on these adverse events, the DSMB determined that the MTD had been achieved and was 60 mg. Two of 23 subjects (9%; mean annualized relapse rate of 0.35) receiving RTL1000 and one of 11 subjects (9%; mean annualized relapse rate of 0.36) receiving placebo had MS exacerbations during the follow-up period; the treating physicians believed that none of these events were treatment related and the DSMB agreed with this assessment.

Adverse events did not lead to subject withdrawal from the study. The most common adverse events in subjects receiving RTL1000 were headache (34.8%), vomiting (30.4%), and nausea (26.1%) and were assessed as treatment related in 26.1%, 26.1%, and 21.7% of subjects, respectively. Subjects receiving placebo had lower frequencies of these side effects: headache (27.3%), vomiting (0%), and nausea (9.1%). While headache, vomiting, and nausea at Grade 1 levels occurred across all dose groups, nausea and vomiting were more likely to be Grade 2 in the 100 and 200 mg dose groups.

#### 3.2.2. RTL1000 Did Not Increase MS-Related Disease Activity

**Figure 2:**
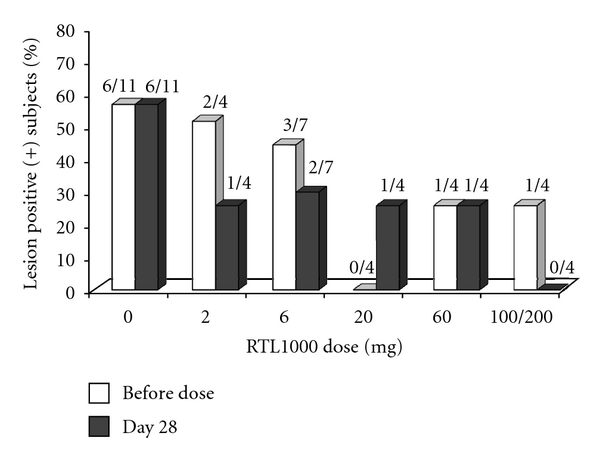
RTL1000 dose and fraction of subjects with gadolinium enhancing lesions at baseline and 28 days after infusion: gadolinium enhancing lesions were scored for each subject at baseline (before dose) and 28 days after infusion of RTL1000 or placebo and the percentage of subjects with ≥1 GAD-enhancing lesion is indicated for each dosing group.

In this study RTL1000 treatment did not worsen MS as assessed by clinical safety endpoints (relapses, EDSS, timed walk, 9-hole peg test) and MRI. As shown in Table 2, the total number of gadolinium enhancing lesions and the number of new gadolinium enhancing and new and enlarging T2 hyperintensities did not increase significantly in any of the cohorts receiving RTL1000. As shown in Figure 2, the frequency of subjects with active MRI scans in the RTL1000 cohorts decreased in three cohorts and remained stable in one cohort following treatment. In the 20 mg cohort, none of the subjects had active scans at baseline and at D28 one subject had developed one gadolinium enhancing lesion. Frequency of subjects receiving placebo with active scans remained stable. Thus, there was no evidence of increased disease activity following RTL1000 administration.

#### 3.2.3. RTL1000 Doses within the MTD Range in Subjects with MS Have Potent Therapeutic Activity at Comparable Doses in Mice with EAE

**Figure 3:**
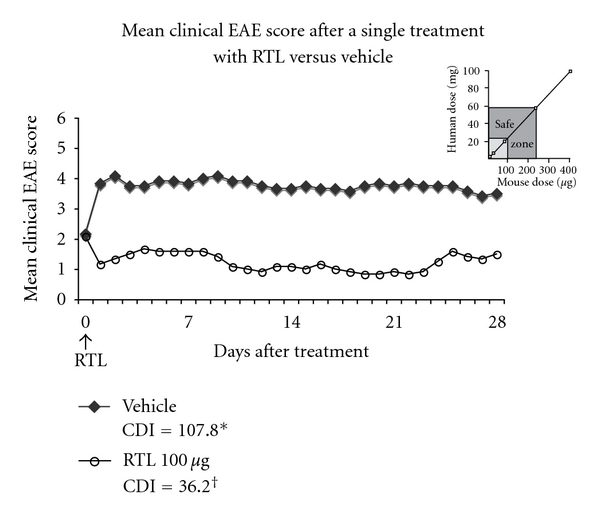
Mouse MOG homologue of RTL1000 treats EAE in DR2 transgenic mice. HLA-DR2 mice were immunized with mouse (m)MOG-35-55 peptide + CFA + Ptx to induce EAE and were treated IV with a single dose of 100 *μ*g of the mouse (m)MOG homologue of RTL1000 (DR2/mMOG-35-55 peptide) or buffer at onset of clinical signs of EAE (indicated as Day 0, corresponding to ~Day 10 after immunization). The mice were scored daily for 28 days for clinical signs of EAE (*n* = 5 mice/group). Reduction in daily scores and cumulative disease scores in RTL versus vehicle-treated mice was significant (*P < 0.05; ^†^ *P* < 0.01, resp.). Inset. Dose comparisons in mice versus humans based on body surface area. Note that the 100 *μ*g dose that is highly effective at treating EAE in mice is equivalent to a ~25 mg dose in humans, well within the safe dose range determined in our study. Figure reproduced in part from Offner et al. [23].

 Based on body surface area measurements, the comparable dose of RTL1000 for treatment of EAE in mice is ~250X less than the dose used in humans [22]. Thus, 60 mg of RTL1000 in MS subjects is comparable to 240 *μ*g in mice (Figure 3 inset). As shown in Figure 3, a single lower dose of 100 *μ*g of the mouse MOG homologue of RTL1000 (equivalent to a 25 mg dose in humans) was sufficient to produce sustained reversal of paralytic signs of MOG-35-55-induced EAE in DR2 transgenic mice over 28 days. These data demonstrate the potent clinical efficacy of RTL1000 homologue in EAE at a dose well within the comparable MTD range in MS subjects.

#### 3.2.4. Treatment with RTL1000 Did Not Induce Immunosuppression

**Figure 4:**
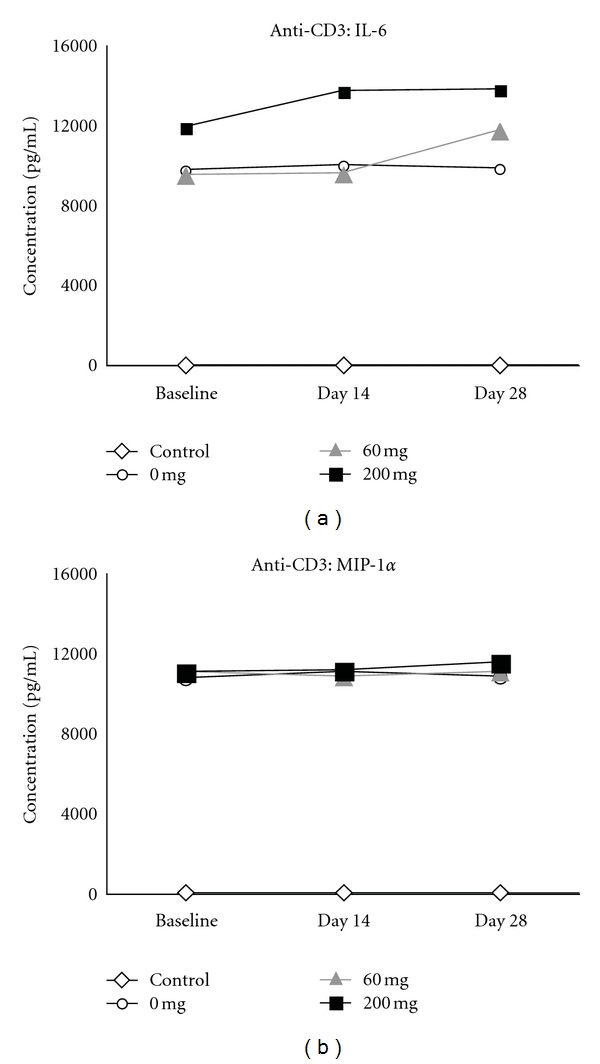
Treatment with RTL1000 did not induce immunosuppression. PBMC collected prior to and 14 and 28 d after infusion of drug from two subjects receiving 60 mg drug, three subjects receiving 200 mg drug and one placebo subject was evaluated for levels of secreted IL-6 (a) and MIP-1*α* (b) in supernatants collected 48 h after stimulation with anti-CD3 mAb or in unstimulated Control cultures. No significant changes were observed at either postinfusion time point.

 Six subjects were evaluated for immunosuppression by assessment of cytokines and chemokines in 48 h supernatants from anti-CD3 mAb-stimulated PBMC cultures prior to and at 14 d and 28 d after infusion of RTL1000 (three subjects receiving 200 mg drug and two receiving 60 mg drug) or placebo (1 subject). No significant reduction was observed in the levels of any of the 12 factors tested, including as examples, IL-6 and MIP-1*α* (Figure 4), suggesting that a single infusion of RTL1000 did not induce immunosuppression.

#### 3.2.5. Treatment with RTL1000 Did Not Induce Significant Changes in Antibody Activity

 ELISA evaluation of sera collected post- versus pre-infusion revealed that the number of MS subjects receiving any dose of RTL1000 who met the criteria for increased levels of IgG and/or IgM antibody to RTL1000 was not significantly different from the number of antibody positive MS subjects receiving placebo (8/20 versus 2/11, *P* = 0.262). Of these, 2 of 8 subjects receiving drug and 1 of 2 receiving placebo had increased antibody responses to DR2, and none had antibody responses to MOG-35-55 peptide. Moreover, the magnitude of IgG or IgM antibody reactivity after infusion versus baseline was not significantly different between the RTL1000 versus placebo-treated groups (Supplementary Figure 1 available online at doi:10.1155/2012/954739).

#### 3.2.6. Pharmacokinetic Profile of RTL1000

**Figure 5:**
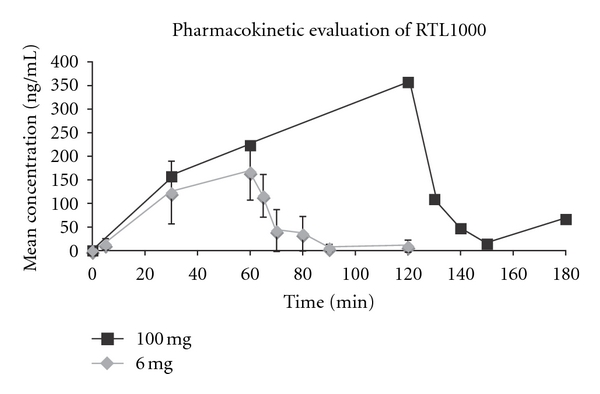
Pharmacokinetic profile of RTL1000. The mean concentration of RTL1000 was assessed in plasma collected from study subjects at the indicated times prior to and after IV infusion of 6 mg (Cohort 2) or 100 mg (Cohort 6) RTL1000. Based on these and other assessments (not shown), the half-life of RTL1000 was determined to be 4.86 ± 2.04 min, with increasing exposure (C_max_ and AUC_last_) observed with increasing dose. Clearance values ranging from 3250 to 44800 mL/min were much greater than hepatic blood flow and indicate that RTL1000 was rapidly eliminated via a nonhepatic mechanism. The high nonphysiological volumes of distribution ranging from 30.8 to 202 liters indicate that RTL1000 is tightly bound to sites not present in plasma.

 PK was determined on plasma from five subjects that received RTL1000 (two received 6 mg; one, 100 mg and two, 200 mg). Subjects receiving 6 mg were infused over 60 minutes and subjects receiving 100 and 200 mg were infused over 120 minutes. Blood plasma samples were collected prior to, during, and after the infusion procedure and were evaluated for RTL1000 levels using sandwich ELISA. The concentration of RTL1000 in plasma is shown in Figure 5 for subjects receiving 6 mg of RTL1000 and for the subject receiving 100 mg. Individual linear regression parameters used to determine the RTL1000 half-lives could be derived in only five of the subjects receiving active drug. RTL1000 was not detected in subjects receiving placebo. Among the five patients receiving drug, the mean ± SD half-life was 4.86 ± 2.04 min with a range of 2.73 to 7.04 min. When the dose was increased from 20 to 60 to 200 mg in Cohorts 3, 4, and 5, the mean C_max_ increased from 3.67 to 12.4 to 70.7 ng/mL, respectively. Total exposure (as assessed using AUC_last_) increased from 35 to 844 to 5090 hr∗ng/mL, respectively. Thus, at these three dose levels, a trend of increasing exposure as assessed by C_max_ and AUC_last_ with dose was observed. Clearance (CL) and volume of distribution could be assessed in only 3 patients (from Cohorts 4 to 6). In these patients, CL ranged from 3250 to 44800 mL/min and volume of distribution ranged from 30.8 to 202 liters. The very high clearance values are much greater than hepatic blood flow and indicate that RTL1000 is rapidly eliminated via a nonhepatic mechanism. The high nonphysiological volumes of distribution indicate that RTL1000 is tightly bound to sites not present in plasma.

## 4. Discussion

This is the first Phase 1 study of any recombinant T-cell receptor ligand in humans. We found that a single infusion of RTL1000 in HLA-DR2^+^ MS subjects was well tolerated at doses of 60 mg or less. Our study also found single infusion of RTL1000 to be safe as there was no indication of immunosuppression or liver enzyme abnormalities in the treated subjects. Subjects receiving RTL1000 did not develop significant antibody responses against RTL1000, DR2 or MOG peptide and there was no evidence of disease activation as detected clinically or by MRI. Importantly, an RTL1000 dose of 60 mg in MS subjects is equivalent to ~240 *μ*g in mice and administration of a single 100 *μ*g dose of a murine RTL1000 homologue was highly effective in treating EAE in DR2 transgenic mice. Thus the maximum tolerated dose of RTL1000 is in a therapeutic range based on EAE studies.

The homologues of RTL1000 designed to treat murine EAE have a remarkable ability to rapidly reverse clinical and histological signs of EAE without causing immunosuppression or toxicity. In a recent report [23], we reviewed preclinical data showing the ability of RTLs to inhibit both targeted cognate and bystander encephalitogenic Th1 and Th17 T-cell specificities in DR2 transgenic [14, 20, 24], C57BL/6 [16, 25], and SJL/J mice [15, 26]. Thus RTL treatment is effective in EAE models induced with three different myelin peptides and involving three different MHC Class II molecules. Importantly, RTLs block entry of inflammatory cells into the CNS [16] and promote remyelination and axonal regeneration in mice with chronic EAE [27, 28]. These studies provide compelling preclinical evidence that RTL1000 therapy in MS has the potential to regulate both MOG-35-55 peptide-specific and bystander T-cells of other specificities, inhibit entry of inflammatory cells into the CNS, and promote remyelination.

RTL1000 is an antigen specific therapy designed to modulate the pathogenic inflammatory response in MS without suppressing the immune system. Because it specifically modulates the immune system, the long-term safety profile of RTL1000 is likely to be better than that of monoclonal antibodies, such as natalizumab, daclizumab and alemtuzumab, and small molecules, such as fingolimod and cladribine, that are immunosuppressive, cause profound lymphocytopenia or alter immunosurveillance within the CNS [29–31]. Antigen specific therapies have the potential to activate MS by stimulating pathogenic T cells as occurred with an altered peptide ligand for MBP-83-99 [32]. We did not observe disease activation in this Phase 1 trial and believe activation is unlikely, as the unique RTL1000 construct ensures that MOG-35-55 peptide-specific T-cells will interact with antigen in the absence of costimulatory molecules. Other antigen-specific therapies, such as oral myelin, intravenous MBP-82-98 peptide [33] and an MBP DNA vaccine [34], have been assessed in MS and were not effective. We believe that RTL1000 is more likely to prove effective because its unique design renders it more efficient at modulating pathogenic immune responses than free peptide or a DNA vaccine. Finally, unlike other antigen-specific therapies, our preclinical studies in EAE suggest that RTL1000 may promote remyelination. Thus RTL1000 represents a promising and novel therapy for MS. 

The PK of RTL1000 is of interest. PK analysis of RTL1000 revealed a dose-dependent increase in exposure and a short half-life of ~5 min only for subjects receiving drug. These data are remarkably similar to our preclinical studies in mice that demonstrated a similar half-life (~10 min, data not shown). The rapid half-life and clearance values and the high nonphysiological volume of distribution suggest that RTL1000 binds to cellular components in blood or possibly to the vascular endothelium. In this regard, RTL binding to mouse antigen-presenting cells inhibited T-cell activation and transfer of EAE [35], and RTL1000 binding to human platelets reduced platelet aggregation and prolonged occlusive thrombus formation in blood [36]. Taken together, these findings suggest that the rapid compartmentalization of RTL1000 to circulating cells and platelets enables inhibitory activity, which may be important to its therapeutic mechanism of action.

The study's limitations include a small sample size, inclusion of a mixture of MS populations, that is, subjects with relapsing remitting as well as secondary progressive subtypes, and relatively short followup. We also only administered a single infusion of RTL1000 and it is possible that disease activation, development of antibodies against RTL1000, DR2 or MOG peptide or other side effects might occur with multiple dosing of RTL1000. We are currently planning to test the safety and potential efficacy of multiple monthly infusions of RTL1000 in a Phase 2 trial.

In summary, our study shows that a single IV administration of RTL1000 is safe and well tolerated up to 60 mg, a dose that is comparable to a clinically effective RTL dose in DR2 mice with EAE. Based on the extensive preclinical testing of RTL in EAE, RTL1000 offers the potential for controlling inflammation without causing immunosuppression and may promote remyelination. Because of its rapid and sustained anti-inflammatory effects and based on the results obtained in the EAE experiments of RTL, a single dose of RTL1000 might be effective in treating relapses of MS. In addition, evaluation of the safety and potential efficacy of monthly infusions of RTL1000 as a long-term treatment for MS may also be warranted given its potent anti-inflammatory effects and apparent ability to promote repair in EAE.

## Supplementary Material

Supplementary Materials includes a figure showing antibody response to RTL1000 components and additional methods.

## Figures and Tables

**Table 1:** Baseline demographics and clinical characteristics.

Characteristic	Placebo	2 mg	6 mg	20 mg	60 mg	200 mg	100 mg	Total
	(*N* = 11)	(*N* = 4)	(*N* = 7)	(*N* = 4)	(*N* = 4)	(*N* = 3)	(*N* = 1)	(*N* = 34)
Female-*N* (%)	8 (73)	4 (100)	5 (71)	3 (75)	4 (100)	2(67)	0	26 (76)
White-not hispanic or Latino-*N*	11	4	7	4	4	3	1	34

Age mean (SD)	52.0 (8.18)	54.3 (2.16)	53.0 (6.30)	58.5 (6.44)	55.9 (7.04)	50.3	38.8	53.2
RRMS *N* (%)	2 (18)	2 (50)	2 (29)	1 (25)	1 (25)	2 (67)	1(100)	11 (32)

SPMS *N* (%)	9 (82)	2 (50)	5 (71)	3 (75)	3 (75)	1 (33)	0 (0)	23 (68)

Number of relapses in the past year (SD)	0.6 (0.81)	0.3 (0.50)	1.0 (1.15)	0.5 (0.58)	0.0 (0.00)	0.3 (0.47)	1	

Time since diagnosis years-mean (SD)	14.5 (10.87)	13.2 (6.04)	13.5 (9.02)	12.1 (6.26)	19.6 (9.42)	7.3 (6.34)	2	

Time since last relapse months-mean (SD)	58 (89.5)	42 (48.0)	48 (68.2)	69 (97.5)	77 (38.4)	32 (26.4)	9	

HLA DR2 homozygous *N* (%)	0	1 (25)	2 (29)	1 (25)	0	1 (33)	0	5 (15)

EDSS score (min, max)	5.41 (3.0, 7.0)	3.38 (2.0, 4.0)	5.71 (3.0, 6.5)	5.00 (4.0, 6.0)	4.88 (3.0, 6.5	3.87 (2.5, 6.0)	2.5	

**Table 2:** Brain MRI outcomes in the placebo and RTL1000-treated subjects.

Cohort (*N*)	Baseline-Gadolinium lesion Total count		Day 28—Gadolinium lesion Total count		Day 28—New Gadolinium lesion count		Day 28—New and Enlarging T2 lesion count	
	Mean	(min, max)	Mean	(min max)	Mean	(min max)	Mean	(min max)
Placebo (11)	1.6	(0, 9)	1.4	(0, 8)	0.6	(0, 2)	0.5	(0, 3)
2 mg dose (4)	0.8	(0, 2)	0.5	(0, 2)	0.5	(0, 2)	0.8	(0, 2)
6 mg dose (7)	0.9	(0, 4)	0.4	(0, 2)	0.0	(0, 0)	0.1	(0, 1)
20 mg dose (4)	0.0	(0, 0)	0.3	(0, 1)	0.3	(0, 1)	0.3	(0, 1)
60 mg (4)	0.3	(0, 1)	0.3	(0, 1)	0.0	(0, 0)	0.0	(0, 0)
100 mg (1)	1.0	(1, 1)	0.0	(0, 0)	0.0	(0, 0)	0.0	(0, 0)
200 mg (3)	0.0	(0, 0)	0.0	(0, 0)	0.0	(0, 0)	0.0	(0, 0)
