# Supplementary material for: Recombinant T-Cell Receptor Ligand (RTL) for Treatment of Multiple Sclerosis: A Double-Blind, Placebo-Controlled, Phase 1, Dose-Escalation Study
Source: Autoimmune Dis. 2012 Apr 5;2012:954739. doi: 10.1155/2012/954739 (PMC3328144; doi:10.1155/2012/954739)
Supplement: Supplementary file 1 — Supplementary Materials includes a figure showing antibody response to RTL1000 components and additional methods. [file 954739.f1.pdf]

Supplemental Information

Methods:

Determination of the Maximum Tolerated Dose (MTD)

The DSMB was empowered to temporarily cease dose escalation and enrollment of the next cohort if any of the following criteria were met: 1) Two or more subjects on active treatment experienced a Grade 1 adverse event and less than two subjects on placebo experienced the same adverse event of Grade 1 or greater severity; 2) One or more subjects on active treatment experienced a Grade 2 adverse event and no subject on placebo experienced the same adverse event of Grade 2 or greater severity; 3) One or more subjects on active treatment experienced a Grade 3 adverse event; 4) A pattern of significant exacerbation of MS was detected in subjects on active treatment, based upon clinical or MRI findings. After the sixth subject in each of the cohort completed the 28-day study, the DSMB met to review safety data from the cohort and evaluate whether a MTD had been reached using the dose escalation/monitoring guidelines and whether enrollment in the next cohort could begin. The next cohort was initiated after authorization of the DSMB.

Antibodies to RTL1000

ELISA assays were performed in 96-well microtiter plates to detect IgG and IgM antibodies specific for RTL1000, MOG-35-55 peptide and HLA-DR2 ("empty" RTL302-5D) based on the criterion that the post-dose serum sample was $\geq$2.5 standard deviations above the baseline mean of the sample collected immediately prior to drug administration. When this criterion was met, the serum sample was considered reactive for antibody against the given protein. Each serum sample was tested in triplicate and on three separate days for a total of nine data points. All sera

were tested at dilutions of 1:50, 1:150 and 1:450. For the ELISA assay, the microtiter wells were coated overnight in coating buffer with antigen. After specific antibody binding to the coating antigen, goat anti-human-horseradish peroxidase conjugate was added to the microtiter plate, followed by TMB chromogenic substrate. The chromogenic reactions were assessed by spectrophotometry and OD values at 450nm. OD values were proportional to the amount of subject antibody and secondary antibody bound to the coating antigen.

RTL1000 Pharmacokinetic (PK) analyses

PK parameters were determined in a subset of subjects. Heparinized plasma samples were drawn from an in-dwelling catheter on the arm opposite the infusion at 5 minutes prior to infusion; 5, 30, and 60 minutes during the infusion; and 5, 10, 20, 30, and 60 minutes following completion of the infusion in the first four cohorts. For the 2-hour infusion (Cohorts 5 and 6), blood samples were drawn at the following times: 5 minutes prior to the infusion; 30, 60, and 120 minutes during the infusion; and 5, 10, 20, 30, and 60 minutes following completion of the infusion.

A sandwich ELISA approach was developed specifically for detection of RTL1000 (the analyte) in patient blood samples. The assay was performed in 96-well microtiter plates. Briefly, wells were coated with a commercial mouse monoclonal capture antibody specific for HLA-DR, -DP, and -DQ (TU39, isotype IgG2a, Becton-Dickinson). After analyte binding to the capture antibody, mouse polyclonal anti-RTL1000 detection antibody (polyclonal reagent produced at OHSU) was added that binds to the analyte. This was followed by IgG1 specific, anti-mouse-HRP conjugate (BD), then by TMB chromogen (KPL). Chromogenic reactions were read by spectrophotometry (OD 450nm) and values proportional to the concentration of analyte were quantified using a standard curve generated by spiking RTL1000 in the subject's own pre-dose plasma. PK metrics, including area under the RTL1000 concentration curve (AUC_{last}) maximum

concentration (C_{max},) half-life ($t_{½}$), clearance (CL) and volume of distribution were estimated on the basis of non-compartmental analysis of plasma RTL1000 concentration-time course data in WinNonlin Version 5.0.1 (Pharsight Corporation). Pharmacokinetic parameters were reported only on baseline-corrected data.

Induction and RTL1000 treatment of EAE

Mice: DR2 transgenic mice [Gonzalez-Gay, 1996] were obtained from Dr. Chella David, Mayo Clinic, Rochester, MN. The mice were bred and housed at the Portland Veterans Affairs Medical Center, Portland, OR, under pathogen-free conditions according to institutional guidelines. Offspring were screened by flow cytometry by examining transgene expression on APC from whole blood obtained from the tail.

Induction of EAE and treatment with the mouse MOG homologue of RTL1000: Transgenic DR2 male and female mice between 8 and 12 weeks of age were immunized subcutaneously at four sites on the flanks with 0.2ml of an emulsion comprised of 200µg human MOG-35-55 peptide in CFA containing 400µg heat-killed Mycobacterium tuberculosis H37RA (Difco, Detroit, MI) as described previously [16]. In addition, mice were given pertussis toxin (Ptx, List Biological Laboratories, Campbell, CA) on Day 0 and Day 2 post-immunization (75ng and 200ng per mouse, respectively). When the clinical EAE scores were $\geq$2, mice were randomized into two groups and received either a single tail vein injection of 100µg of a homologue of RTL1000 (the same DR2 moiety linked to mouse MOG-35-55 peptide that has a P42S substitution compared to human MOG-35-55 peptide) in 100ul of Tris, pH8.5 or 100ul of vehicle (Tris, pH8.5). Mice were scored daily for an additional 28 days according to the following scale: 0 = normal; 1 = limp tail or mild hind limb weakness; 2 = limp tail and moderate hind limb weakness or mild ataxia; 3 = limp tail and moderately severe hind limb weakness; 4 = limp tail and severe hind

limb weakness or mild forelimb weakness or moderate ataxia; 5 = limp tail and paraplegia with no more than moderate forelimb weakness; and 6 = limp tail and paraplegia with severe forelimb weakness or severe ataxia or moribund condition. The mean disease score and standard deviation were calculated on each day. Differences in clinical scores between RTL1000 and vehicle-treated mice were assessed using the Mann-Whitney Test, with significant changes defined as $p \leq 0.05$.

Supplemental Figure 1

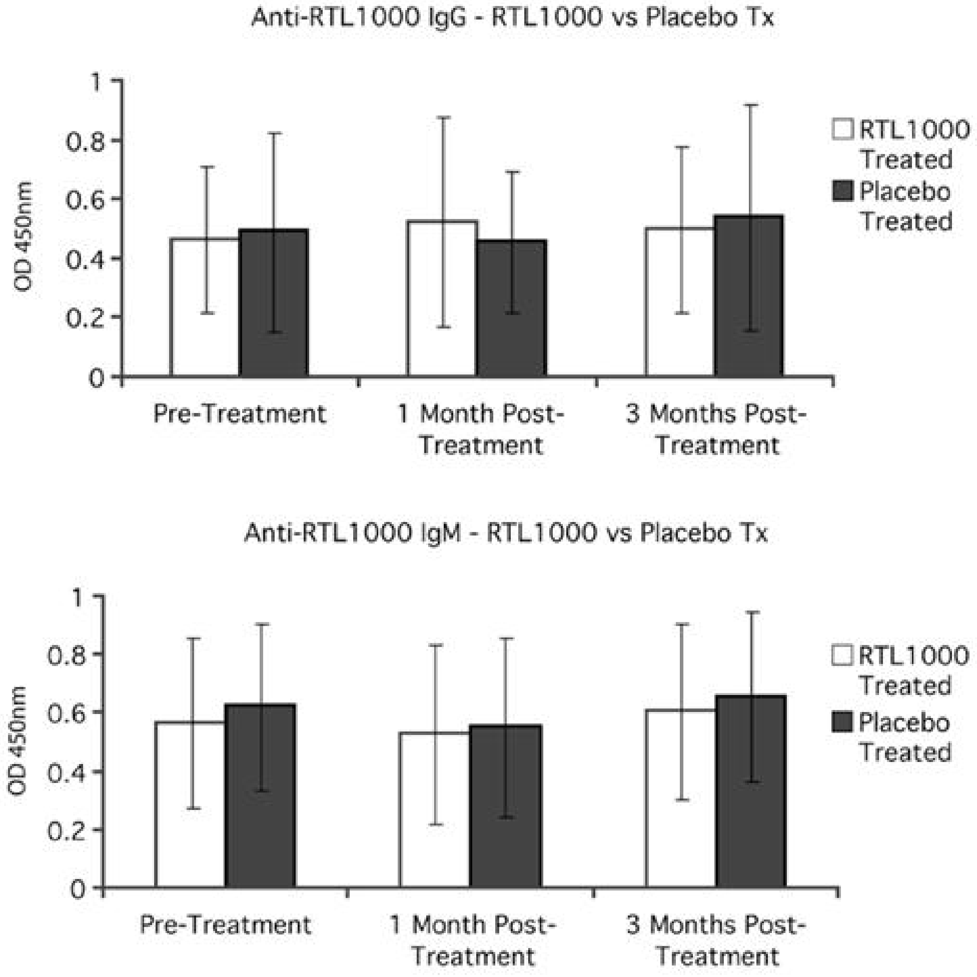

Supplementary Figure 1 Legend: No changes in anti-RTL1000 IgM and IgG serum antibody reactivity after treatment with RTL1000 or placebo: Serum was collected at baseline (Pre-treatment) and at 1 and 3 months post-treatment with RTL1000 (all dose levels included) or Placebo. All subjects in the trial had detectable levels of IgM and IgG antibody reactivity to RTL1000 (1:50 dilution of serum) prior to injection (Pre-Treatment), with no significant changes in serum antibody levels in RTL1000 or Placebo-treated subjects post-infusion ($P>0.5$).
